# Defective DNA polymerase beta invoke a cytosolic DNA mediated inflammatory response

**DOI:** 10.3389/fimmu.2022.1039009

**Published:** 2022-12-23

**Authors:** Shengyuan Zhao, Julia A. Goewey Ruiz, Manu Sebastian, Dawit Kidane

**Affiliations:** ^1^ Division of Pharmacology and Toxicology, College of Pharmacy, The University of Texas at Austin, Dell Pediatric Research Institute, Austin, TX, United States; ^2^ Dept. of Veterinary Medicine & Surgery, University of Texas (UT) MD Anderson Cancer Center, Houston, TX, United States; ^3^ Dept. of Translational Molecular Pathology, University of Texas (UT) MD Anderson Cancer Center, Houston, TX, United States

**Keywords:** DNA polymerase beta, base excision repair, cytosolic DNA mediated inflammatory signaling, PARP inhibitor, interferon type I cytokines

## Abstract

Base excision repair (BER) has evolved to maintain the genomic integrity of DNA following endogenous and exogenous agent induced DNA base damage. In contrast, aberrant BER induces genomic instability, promotes malignant transformation and can even trigger cancer development. Previously, we have shown that deoxyribo-5′-phosphate (dRP) lyase deficient DNA polymerase beta (POLB) causes replication associated genomic instability and sensitivity to both endogenous and exogenous DNA damaging agents. Specifically, it has been established that this loss of dRP lyase function promotes inflammation associated gastric cancer. However, the way that aberrant POLB impacts the immune signaling and inflammatory responses is still unknown. Here we show that a dRP lyase deficient variant of POLB (Leu22Pro, or L22P) increases mitotic dysfunction associated genomic instability, which eventually leads to a cytosolic DNA mediated inflammatory response. Furthermore, poly(ADP-ribose) polymerase 1 inhibition exacerbates chromosomal instability and enhances the cytosolic DNA mediated inflammatory response. Our results suggest that POLB plays a significant role in modulating inflammatory signaling, and they provide a mechanistic basis for future potential cancer immunotherapies.

## Introduction

DNA damage is a biological process that negatively impacts host cells’ genomic integrity and human health (1–4). Cells accrue DNA damage as a result of endogenous metabolic activities or environmental exposures, such as ultraviolet light and chemical mutagens that can promote cancer (5). To ensure genomic integrity, cells have evolved sophisticated mechanisms to repair DNA damage, including base excision repair (BER), which is the predominant repair pathway to process oxidative and alkylating agent derived DNA base lesions (6–10). Further, multiple studies have shown that BER modulates the inflammatory response (11, 12). Mammalian cells harbor two sub-BER pathways that are dependent on the number of oxidized DNA bases to process and the key enzyme involved in the repair process (13). The two sub-pathways are known as short-patch BER (SP-BER) and long-patch BER (LP-BER) (14, 15). SP-BER engages in repairing one nucleotide gaps (16, 17), while the LP-BER involves processing and repairing 2 to 12 nucleotide gaps. Both BER pathways begin as DNA glycosylase recognizes and removes the DNA base lesion. In both pathways, AP-endonuclease 1 (APE1) cleaves the DNA backbone to generate a 3’-OH terminus at the site of damage followed by DNA polymerase beta (POLB), which possesses DNA polymerase and deoxyribo-5′-phosphate (dRP) lyase activities, both of which are known to be important for efficient BER. The dRP lyase activity resides within the 8kDa amino terminal domain of POLB and is responsible for the removal of the 5’-phosphate group (5’-dRP), and subsequently the polymerase domain of POLB adds one nucleotide, leaving a nick which is sealed by DNA ligase I or III (18). While POLB is a major player in SP-BER, LP-BER, involved in processing 2 to 12 nucleotide bases, allows different DNA polymerases such as DNA Pol δ and DNA Pol ε, and other main DNA replication enzymes to conduct strand-displacement DNA synthesis. The displaced single stranded DNA structure or 5’-DNA flap is removed by flap endonuclease I (FEN1) (19) followed by the resulting DNA nicks being sealed by Ligase I or Ligase III (20).

When BER is unable to continue the repair process, there is an accumulation of DNA base damage, single-strand breaks (SSBs) and apurinic/apyrimidinic (AP) sites (21–25). SSBs are converted into double-strand breaks (DSBs) during the S- phase of DNA repalication (26, 27). The BER intermediates such as SSBs and 5’-dRP groups provide the opportunity for poly(ADP-ribose) polymerase 1 (PARP1) to bind and activate poly(ADP-ribose) (PAR) synthesis to facilitate the recruitment of downstream proteins, such as POLB, which fill the gap and XRCC1-Ligase III complex which seals the nick (28, 29). It is possible then that an accumulation of DNA base damage in BER deficient cells could lead to activation of the DNA damage response and modulate an inflammatory response (30, 31). Multiple studies have suggested that DNA repair factors play a role in modulating an inflammatory response (32, 33). Once nuclear DNA integrity is compromised through a deficient DNA repair system or exogenous DNA damaging agents, cells will likely release the DNA into the cytosolic compartment and possibly activate STING signaling and engage an inflammatory response. It is well documented that chronic stimulation of the immune system is critical for tumor promotion and progression (34, 35). One of the key interfaces between defective DNA repair and immunogenicity is the cyclic GMP-AMP synthase/stimulator of IFN genes (cGAS/STING) pathway (33, 36). The cGAS-STING pathway, which senses cytosolic DNA, has been linked to an anti-tumor inflammatory response (37). In this pathway, STING, an endoplasmic reticulum localized protein, is a critical adaptor for the cytosolic DNA sensing pathway (38, 39). Cytosolic double-stranded DNA is sensed by cGAS, leading to activation of the transmembrane protein STING and activation of the transcription factors interferon regulatory factor 3 (mainly IRF3) and nuclear factor kappa B (NF-κB) followed by an upregulation of interferon beta (IFN-β) related genes (40, 41).

Previously, we demonstrated that the human gastric cancer-associated variant of POLB (Leu22Pro or L22P) lacks dRP lyase function *in vitro* and induces replication associated genomic instability and cellular transformation (42). The L22P mutation of POLB lacks dRP lyase activity, which leads to inefficient BER and an accumulation of BER intermediates (21). These intermediates can further block replication fork progression and exacerbate genomic instability (42, 43). Therefore, L22P can serve as a good model to study the interplay between aberrant BER and inflammation in gastric cancer (44). In the present work, we hypothesize that loss of dRP lyase function of POLB enhances cytosolic DNA mediated inflammatory immune signaling through the cGAS/STING pathway. Results from this work show that a novel role of POLB in modulating inflammatory response. We discovered that loss of the dRP lyase function of POLB leads to chromosomal instability and spontaneous upregulation of cytosolic DNA mediated inflammatory response. We also show that targeting PARP1 in dRP lyase deficient cells (L22P variant) exacerbates the release of cytosolic DNA, activates STING signaling, and promotes an inflammatory response. Our study reveals a previously unidentified role of POLB in regulating the cellular inflammatory response thus providing a potential target in a defective BER pathway to enhance an immune based therapy response in the future.

## Material and methods

### Cell lines and materials

We constructed a POLB L22P conditional knock-in mouse model as described previously (21). C57BL/6 Mouse Embryonic Fibroblasts (MEFs) were isolated from embryonic tissue at embryonic day 14.5 (21). Two MEF cell lines isolated from WT and L22P mice were characterized. All animal studies were conducted according to protocols approved by the Institutional Animal Care and Usage Committee of The University of Texas at Austin (protocol # AUP202-00070). Embryos from WT and L22P transgenic mice were isolated at embryonic day 14.5. After the heads, tails, limbs, and most of the internal organs were removed, the embryos were minced and typsinized for 20 min, and then seeded into T-75 cell culture dishes in 10 mL DMEM supplemented with 10% fetal bovine serum (FBS), 1% penicillin/streptomycin, and 1% L-glutamine at 37°C with 5% CO_2_. The cells were split at 1:2 ratios when freshly confluent, passaged two or three times to obtain a morphologically homogenous culture, and then frozen or expanded for further studies.

### Chemicals

To determine whether MEFs are sensitive to exogenous alkylating and oxidative DNA damaging agents, 1-methyl-1-nitrosourea (MNU, Cat. N2939, Spectrum Chemical, New Brunswick, NJ) and H_2_O_2_ (Cat. H1009, Sigma-Aldrich, St. Louis, MO) were dissolved or diluted in water and stored at -20°C before use. Olaparib was purchased from Selleck Chemicals and prepared according to the manufacturer’s protocol (Cat. S1060, Selleck Chemicals).

### Cytoplasmic and whole-cell DNA isolation

Cells were trypsinized and washed with PBS two times before DNA isolation. Whole-cell DNA was isolated using QIAamp DNA Mini Kit (Cat. 51304, Qiagen) according to the manufacturer’s protocol. For cytoplasmic DNA, cells were lysed in hypotonic lysis buffer (10mM HEPES pH 7.4,10mM KCl, 1.5mM MgCl_2_, 0.34M sucrose, 10% glycerol, 0.1% Triton X-100) on ice for 5 mins before centrifuging at 1700g for 5 mins. The supernatant containing the cytoplasmic fraction was collected and centrifuged at 13000g for 10 mins to remove other organelles and incompletely lysed cells. Extraction was validated by Western blot with α-tubulin as the cytoplasmic marker and histone 3 as the nuclear marker. DNA concentration was later quantified using PicoGreen dsDNA assay kit (Cat. P7589, Thermo Fisher) according to the manufacturer’s protocol.

### Alkali comet assay

Alkali comet assay was performed using Comet Assay Single Cell Gel Electrophoresis Assay Kit (Cat. 4250-050-K, Trevigen) according to the manufacturer’s protocol. Cells were mixed with low-melting agarose before plating on comet assay slides for overnight lysis. The next day, chromosomal DNA was denatured under alkali unwinding buffer (pH>13) and underwent electrophoresis (20V) for one hour at 4°C. After drying the slides, DNA was stained with SYBR Gold (Cat. S11494, ThermoFisher) and images were taken with a FITC filter using a Zeiss fluorescence microscope (Zeiss, San Diego, CA, USA) then analyzed by Open Comet Assay using Image J application as described previously (45).

### Abasic site quantification

Genomic DNA was extracted using DNAzol^®^ Reagent (Cat. 10503027, Thermo Fisher) to minimize base loss during sample preparations. DNA was diluted in TE buffer to reach 100ng/µl, and AP sites were measured using AP Sites Quantitation Kit (Cat. STA-324, Cell Biolabs) according to manufacturers’ protocol. Briefly, AP sites were labeled with aldehyde reactive probe (ARP). The probe contains biotin which can be further conjugated with streptavidin-enzyme before performing colorimetric quantification. The standard samples provided in the kit were used to plot a standard curve.

### DNA-PARP-1 crosslinks measurement

Cells were plated and allowed to grow until 70% confluent before Olaparib treatment for 24 hours. Then cells were isolated and lysed with DNAzol. DNA was sheared by passing through a 21-gauge needle and then through a 25-gauge needle, three times each. NaCl was added to reach a final concentration of 4M and incubated at 37°C in a shaking water bath for 20 mins. Urea was then added to reach a final concentration of 4M, and the mixture was incubated for 20 mins in a 37°C shaking water bath. To precipitate DNA-protein crosslinks (DPCs), an equal volume of 100% ethanol was added. The solution was then mixed by inversion followed by the addition of a QIAEX II silica slurry (Cat # 20021, Qiagen). Samples were rocked for 40 mins at room temperature to allow DNA to bind to silica. Silica particles were collected by centrifugation and washed 4 times with 50% ethanol. DPC was eluted from silica by adding 2ml of 8mM NaOH and was incubated at 65°C for 5 mins. The elution process was repeated and the supernatant fractions combined. DPC samples were verified by measuring the DNA concentration. To digest DNA, samples were mixed with digestion buffer (10mM MgCl_2_, 10mM ZnCl_2_, 0.1M NaAc pH=5, 5 units of DNase I, and 5 units of S1 nuclease). The mixture was incubated at 37°C for one hour then at 65°C for 10 mins to stop the digestion. Next, ice-cold trichloroacetic acid (TCA) was added to reach a final concentration of 15% and samples were incubated on ice for one hour to precipitate out DPC proteins. Proteins were pelleted by centrifugation and then washed with 15% ice-cold TCA followed by ice-cold acetone, 2 times each. The pellet was allowed to air-dry and dissolve in RIPA buffer before Western blot.

### Real-Time q-PCR

RNA was extracted using the Trizol/chloroform method and washed with 75% ethanol. cDNA was then immediately synthesized from RNA using High-Capacity cDNA Reverse Transcription Kit (Cat. 4368814, Applied Biosystems). To determine gene expression levels, synthesized cDNA was used as a template for real-time q-PCR using iTaq Universal SYBR Green Supermix (Cat. 1725121, Biorad). Primers are listed in the **Supplementary Table 1**. PCR results were analyzed using 2^–ΔΔ^Ct method.

### Western blotting

Cells were lysed with radioimmunoprecipitation assay (RIPA) buffer supplemented with a protease inhibitor (Cat. 25765800, Sigma Aldrich) and a phosphatase inhibitor (Cat. P5726, Sigma Aldrich). After denaturing the samples at 95°C for 5 minutes, 30μg of each protein sample was separated using SDS-PAGE and transferred onto nitrocellulose membranes (Cat. 1620112, Bio-Rad, Hercules, CA). Next, the membranes were blocked with 5% BSA for 1 hour, and then incubated with primary antibodies against STING (Cat. 13647S, Cell Signaling), IRF3 (Cat. 4302S, Cell Signaling), p-IRF3 (Cat. 4947S, Cell Signaling), TBK1 (Cat. 3013S, Cell Signaling), p-TBK1 (Cat. 5483S, Cell Signaling), β-actin (Cat. 3700S, Cell Signaling), and Vinculin (Cat. 13901S, Cell Signaling) overnight at 4°C. The following day, the membranes were washed with PBST and incubated with anti-mouse (Cat. NXA931, GE Healthcare, Chicago, IL) or anti-rabbit (Cat. NA934V, GE Healthcare) secondary antibody for 2 hours before developing with ECL substrates (Cat. 170506, BioRad). The gel images were captured using Chem-DocXRS image acquisition machine (Bio-Rad).

### Immunofluorescence and micronuclei scoring

WT and L22P MEF cells were cultured in four well chamber slides (Cat # 154453, Thermo Fisher) with complete media. When cell confluency reached 70%, cells were fixed with 3.7% paraformaldehyde (PFA) for 15 mins, followed by permeabilization with 0.5% Triton X-100 for 10 mins. Slides were then blocked with 3% BSA for one hour at room temperature followed by primary antibody incubation overnight at 4°C. Primary antibodies applied include γH_2_AX (1:1000, Cat. 07-164, Millipore), 53BP1 (1:400Cat. Sc-22760, Santa Cruz), α-tubulin (1:400, Cat. 2144S, Cell Signaling), ssDNA (Cat. MAB3299, Sigma), and dsDNA (Cat. ab27156, Abcam). The next day, slides were washed with PBS three times and incubated with secondary antibody for one hour at room temperature. Secondary antibodies applied include Alexa Fluor 488 anti-mouse antibody (Cat. 715-095-150, Jackson immunoResearch Labs) and Texas Red anti-rabbit antibody (Cat. 711-025-152, Jackson ImmunoResearch Labs). Slides were then washed with PBS three times and mounted with mounting media containing DAPI (Cat. H-1200-10, Vector Laboratories) and covered with coverslips. Images were captured using a Zeiss microscope under a 63X objective. The co-localization of γH2AX/53BP1 greater than five foci per per nucleus is considered as the average cut value to identify the difference between different genotypes as well as treated versus untreated group. Micronuclei were identified and quantified as DAPI positive nucleus-shaped particles with diameter smaller than 1/3 of the primary nucleus located nearby.

### Measurement of DNA concentration using PicoGreen

To determine the concentration of DNA isolated from cytoplasm, we applied PicoGreen dsDNA assay kit (Cat. P7589, Thermo Fisher) due to its high sensitivity and accuracy. Cytoplasmic DNA was diluted to 1:10 in 1X TE buffer. The standard curve was prepared using Lambda DNA standard ranging from 10 pg/μL to 1 ng/μL. The standard DNA (100 μL) or DNA samples were mixed with 1X PicoGreen solution of the volume in the dark. The sample mixture was shaken for 5 mins before measuring the fluorescence intensity in a microplate reader at 480nm/520nm (Ex/Em).

### Immunohistology

Gastric tissues from WT and L22P mice were collected and fixed in 3.7% PFA overnight before paraffin embedding. Tissues were then sectioned along the longitudinal axis for immune-histological staining using ImmunoCruz rabbit ABC Staining System (Santa Cruz, sc-2018). Primary antibodies applied include STING (1:200, Cat. 13647S, Cell Signaling), IRF3 (1:200, Cat. 4302S, Cell Signaling), and p-IRF3 (1:100, Cat. 4947S, Cell Signaling). Stained slides were then scanned using Scanscope (Leica Biosystem). For each slide, 5 fields were randomly selected, and the number of positively stained nuclei and total nuclei were counted.

### Statistical analysis

Three independent experiments were performed for immunofluorescence, comet assay, AP site measurement and qRT-PCR. Data were statistically analyzed using Student t-test. Data from more than two study groups were analyzed using two way of ANOVA statstical analysis. Furthermore, the expression of PARP1 and interferon gene correlation was calculated using spearman coefficient with Graph Pad Prism software. Results were considered significant at P< 0.05.

## Results

### dRP lyase deficient POLB cells accumulate genomic instability

**Figure 1:**
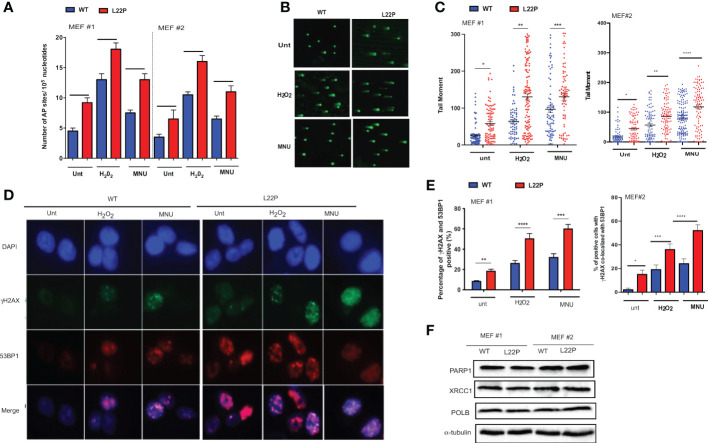
Loss of dRP lyase function causes mitotic dysfunction and telomere crisis. **(A)** Estimated AP sites with and without MNU and/or H_2_O_2_ treatment in WT and L22P cells; The number of AP sites was measured and calculated based upon a standard curve generated using ARP standard DNA solutions as described previously (DNA Damage AP sites assay kit, Colorimetric, Abcam). **(B)** Representative image of single stranded breaks (SSBs) from Comet assay with and without alkylating agent (MNU) and hydrogen peroxide (H_2_O_2_) induced in dRP lyase deficient (L22P) versus proficient (WT) cells; **(C)** Percentage of cells with SSBs in WT versus L22P cells from Comet assay. The data were analyzed based on the paired t-test using GraphPad Prism software. (n=3 independent experiments with at least 100 comets from each groups included for analysis); **(D)** Representative image of co-localization of gH2AX (green) and 53BP1 (red), which represents DSBs; **(E)** Percentage of cells positive for co-localization of H2AX/53BP1 proteins shows DSBs in WT and L22P cells. All images were taken 63x Zeiss microscope from three independent experiments and any cells with >5 foci of γH2AX/53BP1 co-localization per cell were categorized as positive. **(F)** Western bot analysis of BER proteins (PARP1, XRCC1 and POLB) from MEF#1 and MEF#2 cells. Two MEF cell lines (labeled as MEF #1 and MEF #2) were used to generate the data. Two-way ANOVA followed test or student’s test were performed to analayze the data from three independent experiments. P*<0.05, **P<0.01, ***P<0.001, ****P<0.0001.

To determine whether cells with dRP lyase deficient POLB are susceptible to spontaneous and DNA damaging agent induced genomic instability, we characterized two independent MEFs cells (MEF #3 and MEF 2) from each genotype (WT and L22P). First, we examined whether dRP lyase proficient and deficient mouse embryonic fibroblasts (MEFs) cells accumulate base excision repair intermediates including abasic sites (AP sites). AP sites were measured using an AP site assay kit (Colorimetric; Cat. STA-324, Cell Biolabs, USA) that utilizes an aldehyde reactive probe (ARP) reagent that reacts specifically with an aldehyde group, which is the open ring form of an AP site. We observed a significant increase in the enhancement of AP sites in L22P cells versus WT cells (**Figure 1A**; P<0.001). In addition, L22P fibroblast cells significantly harbored spontaneous and exogenous induced single strand breaks (SSBs) compared with WT cells as shown by the formation of longer comet tail moments using an alkali comet assay **(** **Figure 1B, C**). We then considered whether BER intermediates (AP sites and SSBs) contributed to double strand break (DSB) formation with and without DNA damaging agents. We treated WT and L22P cells with MNU or H_2_O_2_ treatment for one hour and examined the colocalization of γH2AX and 53BP1 foci formation (**Figure 1D**). We found that spontaneously and exogenously induced DSBs increased significantly in dRP lyase deficient (L22P) cells versus WT cells (**Figure 1E**; P<0.001). To determine whether or not the presence of L22P variant altered the protein expression of other BER proteins linked to genomic instability, we performed Western blot assay analysis on POLB, PARP1, and XRCC1 and saw no observable difference in protein expression levels between WT and L22P (**Figure 1F**).

### Loss of dRP lyase function increases mitotic dysfunction and accumulation of cytosolic DNA

**Figure 2:**
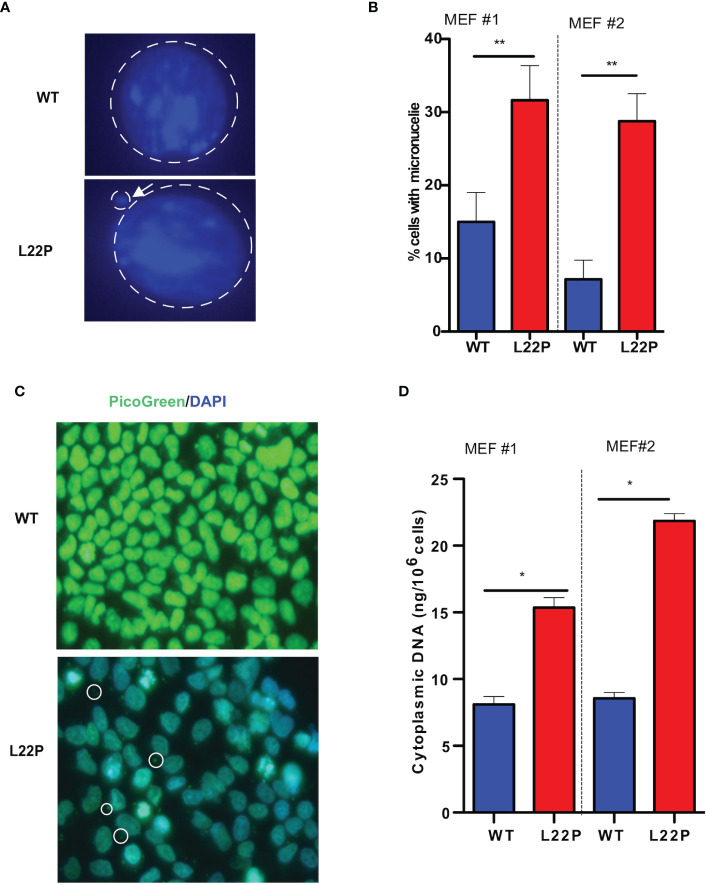
Excessive DNA accumulates in the cytosol of POLB defective cells. **(A)** Representative image of micronuclei formation in dRP lyase proficient and deficient cells; **(B)** Percentage of cells positive for micronuclei; **(C)** Representative image of subcellular localization of cytosolic DNA (bold circle shows the cytosolic DNA, green represents Picogreen stained DNA, and blue represents nuclear DNA stained with DAPI); **(D)** Quantification of cytosolic double-strand DNA (dsDNA) in L22P versus WT cells. Two MEF cell lines (labeled as MEF #1 and MEF #2) were used to generate the data. Data were analyzed using a paired t-test in GraphPad Prism; P*<0.05, **P<0.01.

Previously we have shown that L22P induces chromosomal instability and cytokinesis failure (21). In this study, we examined whether L22P cells enter into mitosis with DNA damage caused by micronuclei formation. We further examined whether the formation of micronuclei could be initiated by errors in chromosome segregation or damaged DNA (**Figure 2A**). The percentage of L22P cells harboring micronuclei was significantly increased versus WT cells (35% versus 13%, P**<0.01; **Figure 2B**). Next, we generated stable MEF cell lines expressing C-terminally HA-tagged POLB-WT or L22P at equal levels to the endogenous WT protein and characterized the micronuclei from each of these lines. We also generated clonal MEF cell lines expressing exogenous HA-tagged human POLB (WT and L22P) at approximately equal levels to endogenous POLB in a tetracycline-repressible manner as described in Supplement Material and Methods. **Supplement Figure 2** shows that MEF cells expressing L22P had increased amounts of micronuclei compared to cells expressing the WT POLB.

Furthermore, **Supplement Figure 2** shows that MEF cells expressing L22P had a significantly higher percentage of cells with DSBs (**Supplement Figure 2B**). Furthermore, the percentage of cells with micronuclei significantly increased in MEF cells expressing HA-Tag L22P-POLB compared to cells expressing the HA- tag WT POLB (**Supplement Figures 2C, D**). To further determine whether BER deficient cells accumulate cytosolic DNA, we examined the localization of cytosolic DNA using PicoGreen immunofluorescence assay using L22P and WT cells. We found that a majority of the L22P cells harbored cytosolic DNA (**Figure 2C** **;** white arrow). To determine whether the aberrant dRP lyase function of L22P leads to an elevated amount of cytosolic DNA, we isolated cytosolic DNA from cytosolic fraction and total DNA from total cell extracts using the Cell Fraction Kit (Cat # ab109719, Abcam) protocol. We performed nuclear and cytoplasmic fractionation of cell lysates followed by DNA precipitation and quantified double-stranded DNA (dsDNA) in the cytoplasmic fractions of L22P and WT cells. The amount of cytosolic dsDNA was significantly higher in L22P cells (16± 3 ng/10^6^ cells) as compared to WT cells (6 ± 0.2 ng/10^6^ cells) **(** **Figure 2D** **;** data presented from two MEFs cells; MEF1 and MEF2). These results clearly demonstrate that aberrant POLB leads to elevated levels of cytosolic DNA.

### Aberrant dRP lyase function of POLB cells activates the cGAS/STING pathway

**Figure 3:**
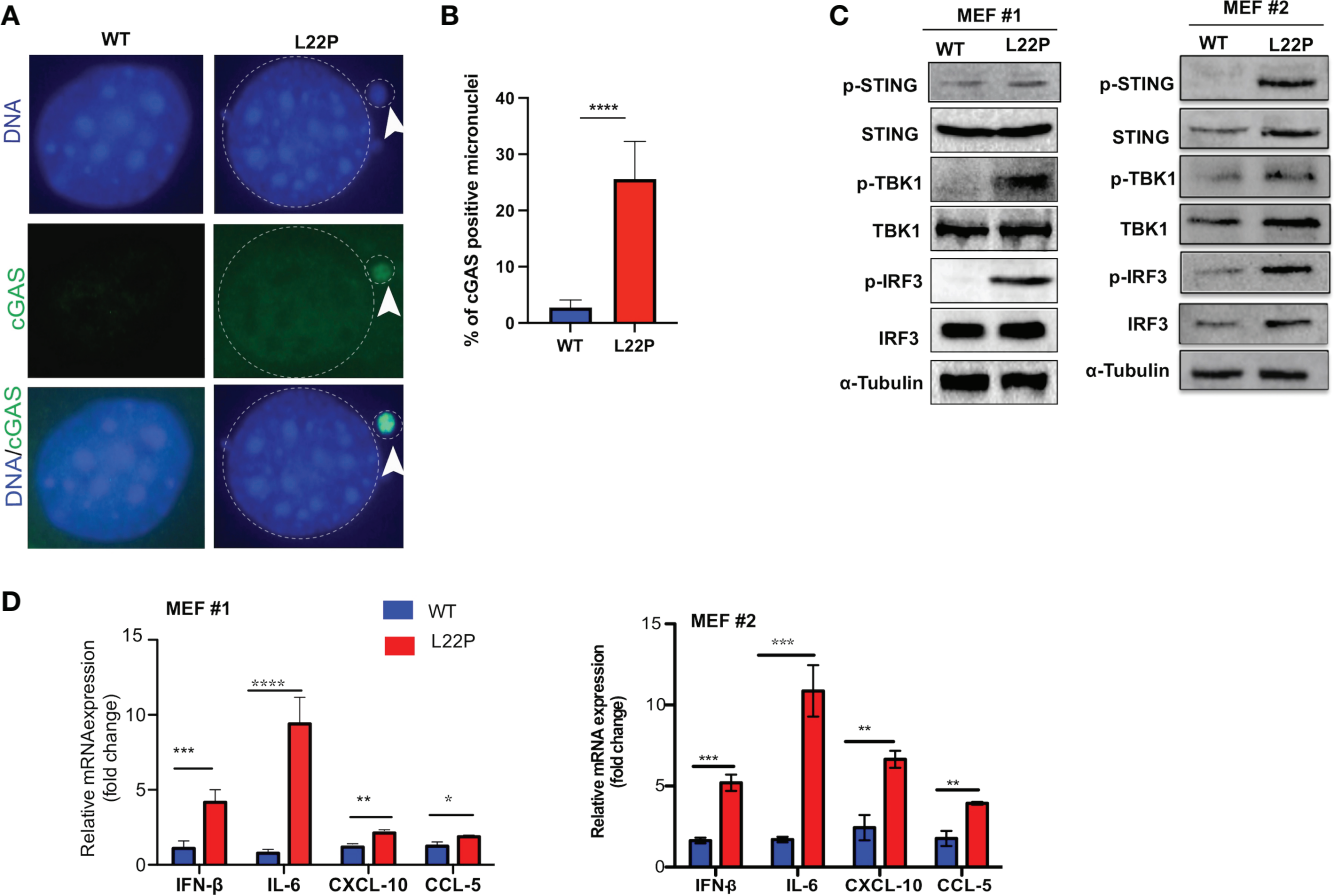
POLB defective cells exhibit cytosolic mediated cGAS-STING activation. **(A)** Representative image localization of cGAS at micronuclei; **(B)** Quantification of positive cGAS localization at micronuclei; **(C)** STING/TBK1/IRF-3 signaling pathway activation detected with Western blot of protein extract from WT and L22P cells. Anti-STING/anti-Phospho-STING (Ser366); IRF3/p-IRF3 (ser385); TBK1/P-TBK-1 (Ser172) antibodies were used to detect the activation of cGAS/STING dependent pathway. Two MEF cell lines (labeled as MEF #1 and MEF #2) were used to generate the data; **(D)** Fold change in mRNA expression of type I interferon cytokines measured using RT-qPCR in dRP lyase deficient (L22P) versus proficient cells (WT). Data were analyzed using a paired t-test in GraphPad Prism; P*<0.05, **P<0.01, ***P<0.001, ****P<0.0001.

Micronuclei arise following the mis‐segregation of broken chromosomes during mitosis (46–48) and have recently been described as platforms for cGAS/STING‐mediated immunity activation following DNA damage (47–49). We found that unrepaired DSBs trigger mitotic dysfunction (micronuclei formation) **(** **Figures 2A, B**). In addition, to determine whether cGAS localization in micronuclei is a general phenomenon in L22P cells, we transfected WT and L22P MEFs cells with pMSCVpuro-GFP-cGAS or stably expressing GFP-cGAS plasmids (generous gift from Dr. Andrew P. Jackson & Dr. Martin A. Reijns, MRC, UK) and examined the colocalization of cGAS at the micronuclei. As seen in **Figure 3A**, we found that cGAS strongly colocalized with micronuclei in L22P cells. In addition, the percentage of DNA sensor (cGAS) positive micronuclei was significantly increased in cells with the dRP lyase deficient POLB (27%) (**Figure 3B**), suggesting that nuclear DNA (nDNA) released from micronuclei may be an important danger signal to elicit an inflammatory response, functioning in an immune-stimulatory role triggering downstream factors of the STING-TANK binding kinase 1 (TBK1)-IRF3 inflammatory signaling axis. To determine whether L22P induced micronuclei trigger STING signaling activation, we examined which downstream cGAS/STING pathway proteins were activated by Western blot analysis. We found that STING-TBK1-IRF3 signaling pathways was activated in dRP lyase deficient cells [as seen by phosphorylation of STING at Ser366 (pSTING); p-TBK1 (Ser172) and p-IRF3 (Ser385)] (**Figure 3C**; from MEF1 and MEF2 cell lines). Moreover, to examine whether micronuclei formation induced in L22P cells might stimulate a cytokine response, we measured the levels of mRNA expression of type I interferon cytokines in WT versus L22P cells and found that interferon beta 1 (IFNβ), C-X-C motif chemokine ligand 10 (CXCL10), C-C motif chemokine ligand 5 (CCL5) and interleukin 6 (IL-6) were significantly increased in L22P cells versus WT (**Figure 3D** **;** P***<0.001; P***<0.001). Overall, this data suggested that a normally functioning POLB is required to prevent a spontaneous immune response.

### Targeting PARP1 exacerbates mitotic dysfunction and enhances cytosolic DNA mediated inflammatory signaling in dRP lyase deficient cells

**Figure 4:**
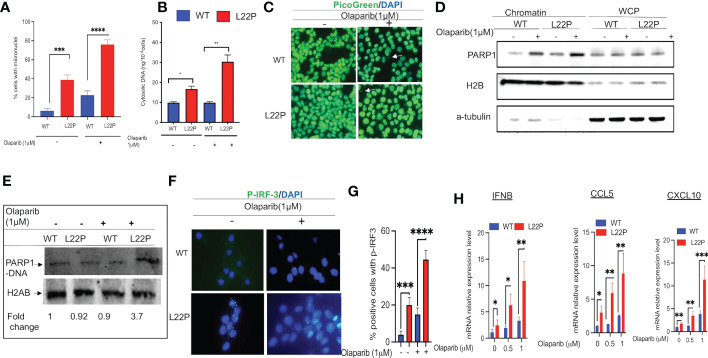
Targeting PARP1 [Olaparib (PARPi)] increases a defect in chromosomal segregation and promotes an inflammatory response. **(A)** Percent of positive cells with micronuclei after Olaparib treatment for 24 hours in L22P versus WT; **(B)** Quantification of cytoplasmic DNA from dRP lyase proficient and deficient cells; **(C)** Representative image of cells stained with Picogreen and DAPI to show cytoplasmic DNA with Olaparib and without in dRP lyase proficient and deficient cells (white arrow); **(D)** Chromatin association of PARP1 in Olaparib treated and untreated dRP lyase proficient and deficient cells; **(E)** PARP1-DNA complex analysis using dRP lyase proficient and deficient cells with and without Olaparib treatment; **(F)** Subcellular localization of p-IRF3 in dRP lyase proficient versus deficient cells with and without Olaparib treatment; **(G)** Quantification of P-IR3 positive nuclei with Olaparib treated and untreated dRP lyase proficient and deficient cells; **(H)** mRNA expression of type I interferon genes using RT-qPCR (IFNB, CCL5 and CXCL10) from WT and L22P cells with and without Olaparib treatment. Data were analyzed using a paired t-test in GraphPad Prism; P*<0.05, P**<0.01, P***<0.001, P****<0.0001.

PARP1 is known to be activated in response to DNA damage and is responsible for the synthesis of the majority of poly(ADP-ribose) (PAR) following genotoxic stress (50, 51). In addition, PARP1 modulates different DNA repair pathways, mitosis, gene expression and cell death (51–61). Previously, we have shown that PARP1 inhibitor exacerbates genomic instability in dRP lyase deficient cells (42). PARP1 inhibitor-mediated trapping of PARP1 on DNA lesions appears to be influential for the DNA-STING immune response, as the extent of PARP1 trapping correlates with the magnitude of immune signaling (62). To determine whether blocking PARP1 enhances a DNA sensor mediated inflammatory response in dRP lyase deficient cells, L22P MEF cells were treated with the PARP1 inhibitor Olaparib (1μM). We then examined any resulting mitotic dysfunction and cGAS/STING downstream signaling cytokines. We found that 80% of L22P expressing cells harbored micronuclei versus WT cells (20%) after Olaparib treatment (**Figure 4A**; P****<0.001). Moreover, Olaparib treatment significantly induced cytosolic DNA in L22P cells (30ng/10^6^ cells) versus WT (10ng/10^6^ cells) (**Figure 4B**; P*** <0.001) and Olaparib treatment in dRP lyase deficient cells enhanced the cytoplasmic DNA localization (**Figure 4C**). Furthermore, we examined whether Olaparib treatment increased chromatin association of PARP1 in L22P cells as compared with treated WT and untreated L22P. We found that Olaparib treatment did induce chromatin associated PARP1 in dRP lyase deficient cells (**Figure 4D**). We also measured PARP1 trapping in dRP lyase deficient cells using a DNA silica assay (see Materials & Methods section) and found that PARP1 trapping significantly increased 3.7 fold in dRP lyase deficient cells (**Figure 4E**). In support of this observation, we stained dRP lyase deficient and WT cells with a primary antibody against p-IRF3 (at Ser385) and detected that the translocation of p-IRF3 to the nucleus significantly increased in L22P cells treated with Olaparib versus WT (**Figures 4F, G**; P**<0.01). Furthermore, the mRNA expression of type I interferon response cytokines/chemokines (IFNβ, CXCL10 and CXCL5) significantly increased in Olaparib treated L22P cells versus WT (**Figure 4H**; P<0.001). Next, we considered the relationship among PARP1 and interferon-stimulated genes (ISGs) at the transcriptional level in cancer patients by analyzing the transcriptome profiles in The Cancer Genome Atlas (TCGA) database. Our analysis indicated that PARP1 expression was negatively correlated with the expression of ISGs (IRF7 and ISG15) in human stomach cancer (n = 407 samples, P < 0.01), which is consistent with our *in vitro* study observations (**Supplement Figure 2**).

### dRP lyase deficient POLB triggers cytosolic DNA mediated chronic inflammation in L22P mice

**Figure 5:**
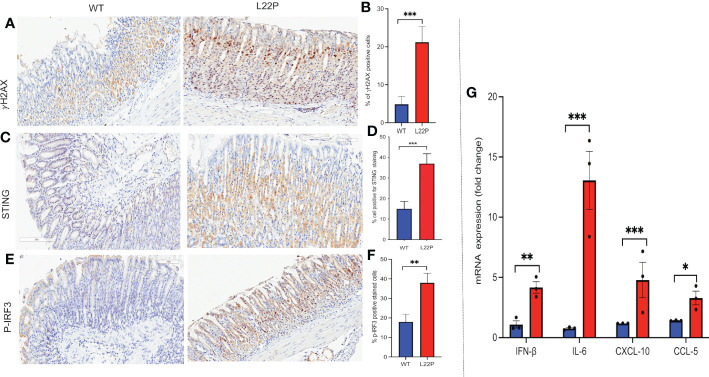
cGAS/STING activation in dRP lyase deficient mice. **(A)** Immunohistochemistry staining of stomach tissue section with DSB marker (gH2AX) in dRP lyase deficient (L22P) and WT mice; **(B)** Percentage of cells positive for gH2AX; **(C)** Immunohistochemistry staining of STING on stomach tissue of dRP lyase deficient versus proficient WT mice; **(D)** Percent of cells positive for STING **(E)** Immunohistochemistry stain of Ph-IRF3 localization in stomach tissue section of L22P versus WT mice **(F)** Percent of cells positive for ph-IRF3 **(G)** Quantification of mRNA cytosolic DNA-STING signaling mediated cytokines expression using qRT-PCR from stomach tissues derived from dRP lyase deficient and proficient WT mice. Data were analyzed using student t-test in GraphPad Prism; P*<0.05, P**<0.01, P***<0.001.

STING has recently been identified as one of the critical adaptors for sensing cytosolic DNA, followed by the phosphorylation of IRF3 and subsequent production of type-I IFN and IL-6 (63). Previously, we have found that L22P induces an accumulation of DSBs and inflammation in mice (21). To gain further insight into how spontaneous DNA damage in L22P mice drives cytosolic mediated inflammatory response, we studied the stomach of L22P and age-matched WT littermate control mice. We observed that the stomach tissue from L22P mice stained with an antibody against H2AX showed a significant percentage of positively stained cells as compared with stomach tissue derived from WT mice, which indicates an increased level of genomic instability in the L22P mice (**Figures 5A, B**). We then explored the expression levels of cGAS-STING pathway proteins using immunochemistry and found that the L22P mice stomach tissue had significant changes in both STING (**Figures 5C, D**) and p-IRF3 protein levels as well as subcellular localization (**Figures 5E, F**). Furthermore, the mRNA expression of interferon type-I cytokines including IFNβ, CXCL10, and CCL5 significantly increased in the stomach tissues of dRP lyase deficient mice versus WT mice (**Figure 5G**; P***<0.001).

## Discussion

We report in this paper that POLB with a defective dRP lyase function plays a major role in cellular mitotic dysfunction and increased genomic instability. In particular, our data show that dRP lyase deficient cells harbor unrepaired BER intermediates such as apurinic/apyrimidinic (AP) sites and single-stranded DNA breaks (SSBs) that are potentially converted into DSBs. AP sites are among the most frequent spontaneous lesions in DNA. AP sites are replication-blocking lesions that could result in the accumulation of DSBs, leading to chromosomal fragmentation and genomic instability if not repaired in an accurate and timely manner (64, 65). In addition, cleavage of AP sites by AP endonucleases or AP lyases generates DNA single-strand breaks (SSBs) with 5’- or 3’-blocked ends (65). It has been previously reported that an accumulation of oxidative stress related DNA damage eventually causes replication stress in BER deficient cells (66). Our study supports that finding and shows that exposure to oxidative and alkylating DNA damaging agents exacerbates DNA damage and aberrant mitotic features in dRP lyase deficient cells. This observation aligns with our previous results demonstrating that POLB dRP lyase deficiency increases replication associated DSBs (42). Furthermore, an elevation of micronuclei formation is commonly observed in dRP lyase deficient cells, a sign of spontaneous genomic instability. Our previously published data have shown that POLB dRP lyase deficient cells harbor mis-chromosomal segregation phenotypes and cytokinesis failure that derives from unrepaired DSBs progressing through mitosis (21). In line with this result, deficiency in several DNA repair pathways is associated with an increased frequency of micronuclei (67, 68). Importantly, other studies have demonstrated the molecular mechanism of micronuclei formation in cells following unrepaired DNA damage progressing through mitosis (48, 69).

Micronuclei formation is a consequence of irreversible nuclear envelope collapse, which arises frequently in cells due to defective nuclear lamina organization (70). It is well documented that micronuclear DNA is particularly susceptible to DNA damage, leading to chromothripsis (46, 71). We wanted to better understand how dRP lyase deficient cells with micronuclei may contribute to a release of cytosolic DNA that may play a predominant role in triggering cGAS/STING signaling. As shown in **Figure 2**, we analyzed the cytosolic subcellular localization of dsDNA and cytosolic DNA concentration measurements from cell extracts and found that POLB dRP lyase deficient cells accumulate cytosolic DNA which potentially serves as a danger associated molecular pattern. Our results show that a loss of nuclear genomic integrity in POLB dRP lyase deficient cells enables the cells to accrue cytosolic DNA. Similarly, other studies have shown that homologous recombination repair genes such as RPA and RAD51, which support genome stability during replication, were shown to prevent the accumulation of cytosolic DNA (72). In addition, several DNA damage response genes (e.g. ATM and DNA sensor MRE11) were found to prevent an accumulation of cytosolic DNA (32, 73). It is possible that micronuclei rupture results in immunostimulatory cytosolic DNA being recognized by cGAS, thus activating immune surveillance (48), and possibly leading to an inflammatory immune response that is known to be triggered by cytosolic DNA (74). The localization experiment as shown in **Figure 3** demonstrated that a cGAS significantly localized to micronuclei in POLB dRP lyase deficient cells. In support of this observation, Mackenzie KJ, et al. have reported that cytosolic DNA accumulation is a result of genomic instability and triggers a cGAS/STING-dependent interferon response (48), which our observation support. Another study has shown that inactivation of the DNA repair genes BRCA2 results in cGAS-positive micronuclei which also triggered a cGAS-STING dependent interferon response (75). Moreover, defects in cellular DNA damage response can induce cytosolic DNA which also has been linked to a cGAS-STING mediated immune response (39, 76). Additionally, DNA structure-specific endonuclease MUS81, which cleaves DNA structures at stalled replication forks, also mediates a STING-dependent activation of immune signaling (77). Similarly, our findings highlight the involvement of DNA polymerase beta in cytosolic DNA mediated inflammatory response.

Targeting BER factors may increase cytosolic DNA and enhance cGAS-STING signaling which could increase the immunogenicity of a tumor’s microenvironment. A recent publication has shown that POLB deficiency triggers cytosolic DNA mediated cGAS-STING signaling pathway activation in immune cells with autoimmune disease (78). Previously, we have shown that treatment with PARP inhibitor increases replication associated DSBs in dRP lyase deficient cells during S-phase of the cell cycle (79), which suggests that dRP lyase deficient cells accumulate 5’-dRP groups, which are critical for interaction with PARP1. Mechanistically, PARP inhibitor engages PARP1 to form a covalent bond with 5’-dRP groups and blocks BER (79) or hinders the BER process (80). Results from our study demonstrate that treatment of dRP lyase-defective cells with PARP1 inhibitor (Olaparib) increased mitotic defects and resulted in an elevated number of micronuclei. Those dRP lyase deficient cells with unrepaired DSBs will likely progress into mitosis, leading to mis-segregation of a chromosome resulting in micronuclei formation. Our data are in agreement with similar findings on the impact of PARP inhibitor causing mitotic defects such as chromosome misalignment, anaphase DNA bridges, lagging chromosomes, and micronuclei formation (81). Further, in this work we report that PARP1 inhibitor treated dRP lyase deficient cells accumulate cytosolic DNA and exhibit a significant increase in the amount of PARP-DNA complexes as well as chromatin associated PARP1 (**Figure 4**). As a consequence, targeting PARP leads to elevated levels of cytosolic DNA mediated cGAS-STING signaling. Our results support another previously published finding that PARP-trapping is critical for the induction of immune signaling (62). In addition, our *in vivo* data show that there is an increase in the protein expression of STING and p-IRF3 in the stomach tissue of POLB dRP lyase deficient mice. From our histological analysis, it seems that the parietal cells, which are found in the gastric glands of the stomach fundus and body, are the major target of DNA damage and IRF3 phosphorylation. Further, we show that cytokine mRNA expression significantly increased in dRP lyase deficient mice stomach tissues versus WT mice stomach tissue. These results suggest that the normal function of POLB is required for maintenance of immune homoeostasis.

Overall, our results suggest that normal function of POLB is critical to suppress cytosolic DNA mediated cGAS-STING activation. Further, PARP inhibitor treatment exacerbates cGAS-STING signaling in POLB defective cells. Our data demonstrate that PARP inhibition could be used to further increase micronuclei formation and thereby force activation of the subsequent cGAS-STING-mediated inflammatory response. It is possible that other potential cytosolic nucleic acid receptor pathways are likely activated and trigger multiple signaling cascades in dRP lyase deficient cells to trigger type I interferons and activation of TBK1, IRF3. Many studies have shown that Type I IFNs,TBK1 and IRF3 are activated by toll-like receptors (TLRs) and cytosolic nucleic acids (RNA and DNA) sensors such as RIG-I-like receptors (RLRs) (82–85). We hope that our observations may open up new opportunities to build on this existing work and lead to an understanding of how the various cytosolic nucleic acid receptors enable dRP lyase deficient cells to induce type I interferons and pro-inflammatory cytokines. Furthermore, our study lays a foundation for future exploration into whether PARP1 inhibitor treatment might provoke inflammatory signaling and enhance immune checkpoint inhibitor treatment in BER deficient cancer patients.

## Data availability statement

The original contributions presented in the study are included in the article/**Supplementary Material**. Further inquiries can be directed to the corresponding author.

## Ethics statement

The animal study was reviewed and approved by The University of Texas at Austin, IACUC.

## Author contributions

JG performed *in vitro* experiments and participated in data analysis; SZ performed data analysis, data interpretation, and manuscript writing; MS provided pathological analysis of the data and provide pathological interpretation. DK performed data analysis, interpretation, and manuscript writing. All authors contributed to the article and approved the submitted version.
