# Supplementary material for: Defective DNA polymerase beta invoke a cytosolic DNA mediated inflammatory response
Source: Front Immunol. 2022 Dec 23;13:1039009. doi: 10.3389/fimmu.2022.1039009 (PMC9823925; doi:10.3389/fimmu.2022.1039009)
Supplement: Supplementary file 1 [file DataSheet_1.pdf]

Supplement Material and Methods

Mammalian cell lines and cell culture : For cell culture experiments, mouse Pol β cDNA with a C-terminal hemagglutinin (HA) tag was cloned into the pRVYTet-Sis retroviral vector as described (1, 2) . The L22P variant was introduced into the mouse WT (22Pro) Pol β cDNA sequence using site-directed mutagenesis (Stratagene) following the manufacturer's protocols. The GP2-293 virus packaging cell line (Clontech) was used for retrovirus preparation. These cells were maintained in Dulbecco modified Eagle's medium (Invitrogen) supplemented with 10% fetal bovine serum (Invitrogen), 1% L-glutamine (Invitrogen), 1% penicillin-streptomycin (Invitrogen) and 1 mM HEPES (Invitrogen). Mouse Pol β WT and L22P constructs were packaged into retrovirus using the GP2-293 packaging line. pRVYTet and pVSV-G plasmids were co-transfected into GP2-293 cells using standard calcium phosphate transfection, cells were grown for 72 hours, and retrovirus was harvested.

Transfection, Infection, and Expression Analysis: To infect MEFs WT cells , cells were grown to approximately 30% confluence and infected with retrovirus in the presence of 4 μg/ml polybrene. Cells were incubated overnight in fresh media with 4 μg/ml polybrene. For selection of pools, cells were split 1:3 the day after infection and cells with the integrated construct were selected with 100 μg/ml hygromycin B for the MEFs. Clonal cell lines were propagated in the presence of 100 μg/ml hygromycin B. Expression of exogenous HA-tagged Pol β was verified by Western blot. Cells were passed in parallel in the presence or absence of tetracycline. Approximately 80–90% confluent cells were harvested by scraping with hot SDS Loading Buffer (50 mM Tris pH 6.8, 100 mM DTT, 2% SDS 10% glycerol). Lysates were boiled for 10 minutes and run

on a 10% acrylamide SDS-PAGE gel. Proteins were transferred to nitrocellulose membrane using a semi-dry transfer apparatus and probed using monoclonal mouse anti-Pol β antibody (Abcam #1831).

References

1. Lang T, Dalal S, Chikova A, DiMaio D, Sweasy JB. The E295K DNA polymerase beta gastric cancer-associated variant interferes with base excision repair and induces cellular transformation. Mol Cell Biol. 2007;27(15):5587-96.

2. Sweasy JB, Lang T, Starcevic D, Sun KW, Lai CC, Dimaio D, et al. Expression of DNA polymerase {beta} cancer-associated variants in mouse cells results in cellular transformation. Proc Natl Acad Sci U S A. 2005;102(40):14350-5.

Supplement Figure 1

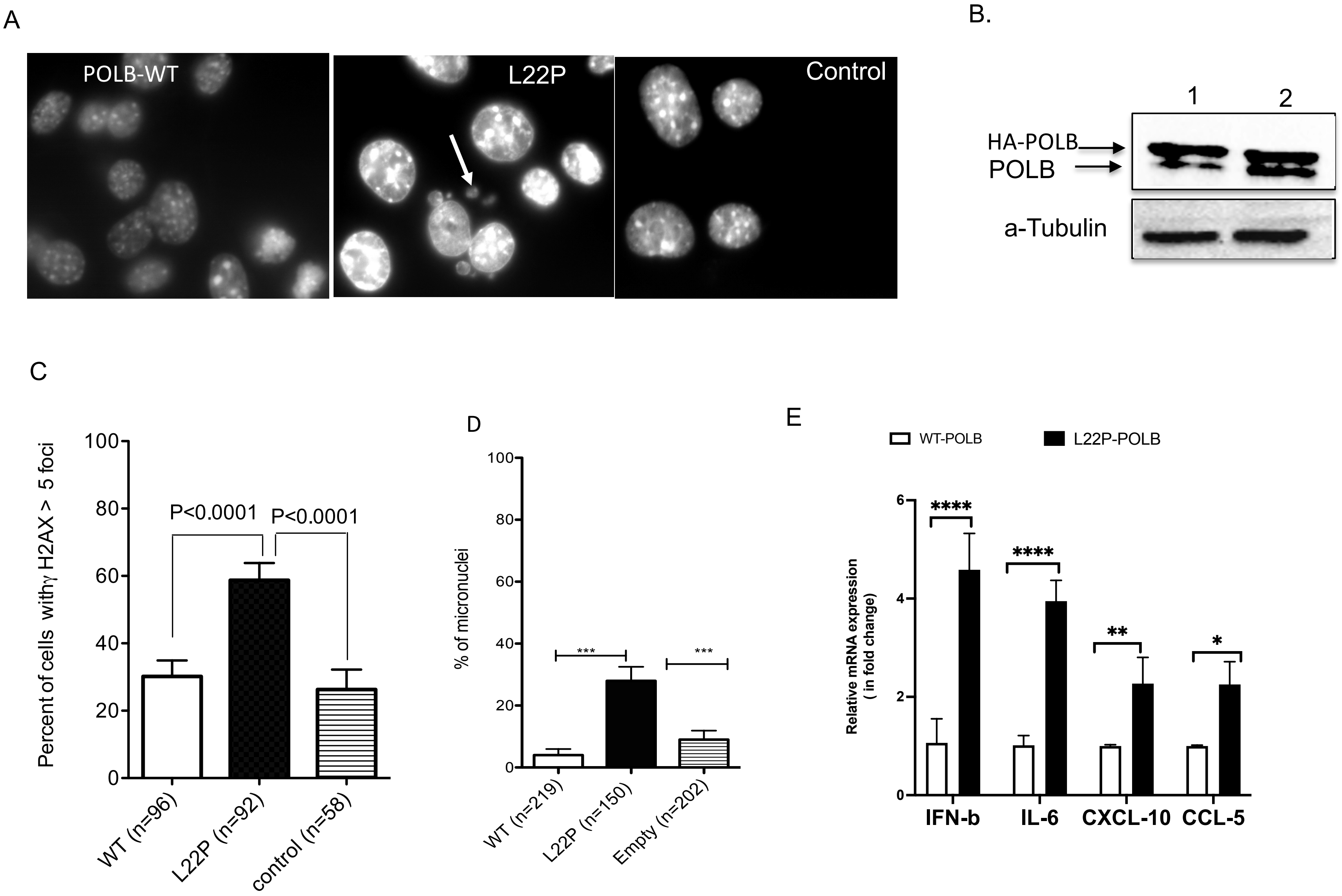

Supplement Figure 2

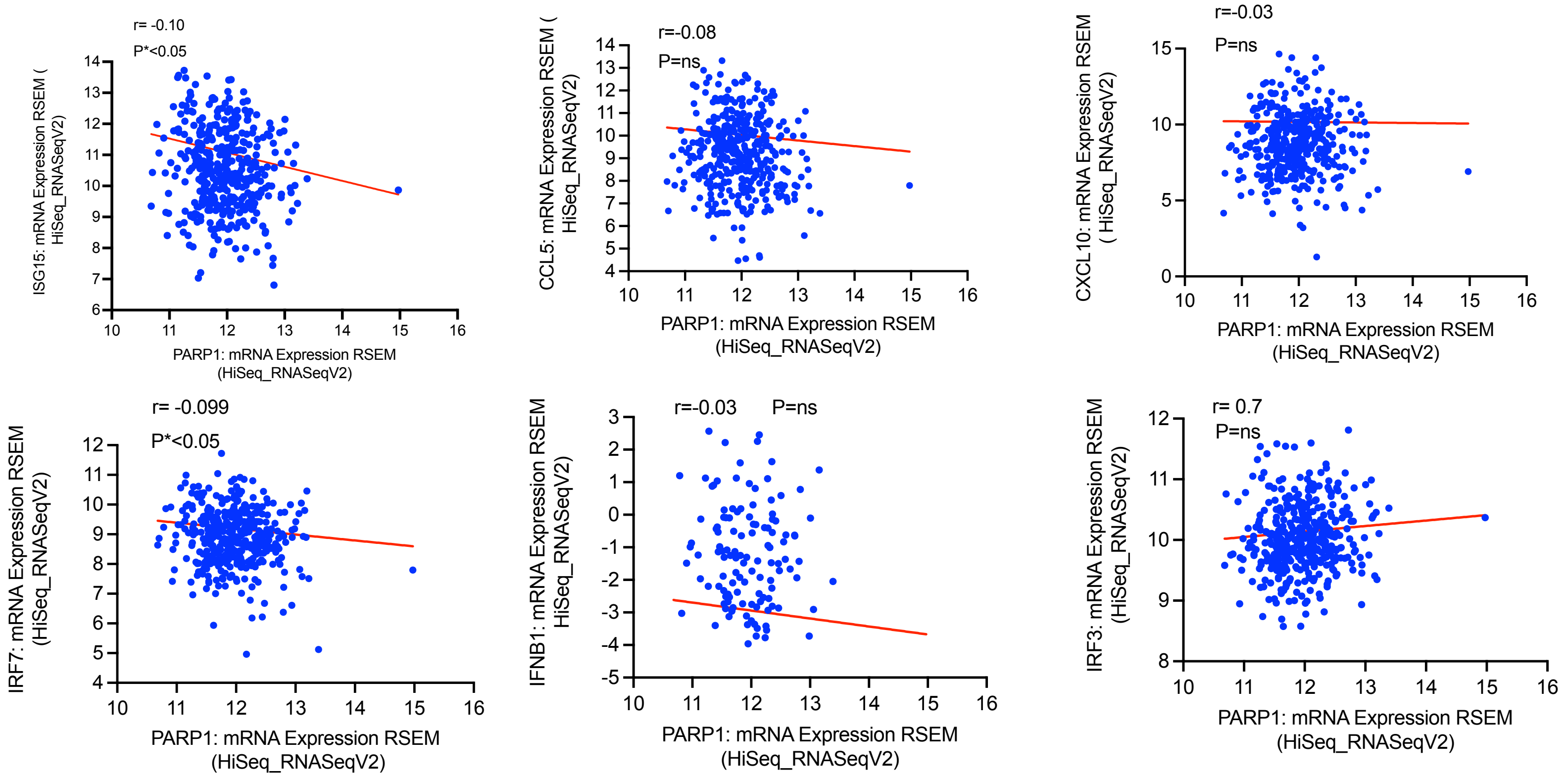

Supplement Figure 1. Expression of L22P in WT cells induces genomic instability and dominant negative cells A) Exogenous expression of L22P Pol β expression in cell lines. A. Representative western blot for Pol β expression in MEFs cells. Lane 1 is from the WT line and lane 2 is from the L22P cell line; B) The percent of cells with γH2AX/53BP1 positive in MEFS cells expressing WT-POLB and L22P-POLB; C) The percent of micronuclei in POLB-WT, L22P-POLB and control (empty plasmid); C) Image represent micronuclei from POLB-WT and L22P cell line; E) Type I interferon cytokine profile of the POLB-WT, L22P-POLB expressing cells. Two-tailed t-tests and two-way analysis of variance (ANOVA) were used as appropriate to determine whether the mean of each cell line was different from the empty vector cells. Bonferroni's post hoc test was used to determine significant differences between the means of each group. All statistics were performed using GraphPad Prism version 5 (GraphPad Software, San Diego, CA). Data are represented as mean ± SEM.

Supplement Figure 2. PARP1 expression positively correlated with innate immune signaling genes. TCGA gastric cancer data sets were pulled from cBioProtal (http://cbioportal.org). Since PARP1 is the primary target of interest for this analysis, only individuals with valid RNA Seq V2 RSEM data for PARP1 were included (n=407, sample from patients) from TCGA Pan Cancer Atlas. The correlation of the change in PARP1 expression in gastric cancer and innate immune signaling inflammatory cytokines (IRF7, ISG15, CCL5 CXCL10 and IRF3) were analyzed using Graph Pad prism. Data were analyzed using Spearman correlation analysis**.** *R* and *P* represent

Spearman correlation coefficients and 2-tailed *P* values. P*<0.05 represent significance and ns represent non significance difference.

Supplement Table 1. List of primer for measuring mRNA expression using for RT-PCR
