# Supplementary material for: Defective DNA polymerase beta invoke a cytosolic DNA mediated inflammatory response
Source: Front Immunol. 2022 Dec 23;13:1039009. doi: 10.3389/fimmu.2022.1039009 (PMC9823925; doi:10.3389/fimmu.2022.1039009)
Supplement: Supplementary file 2 [file Table_1.doc]

| Gene | Forward (5’-3’) | Reverse (5’-3’) |
| --- | --- | --- |
| mCCL5 | TGCAGAGGACTCTGAGACAGC | GAGTGGTGTCCGAGCCATA |
| mIFN-ß | AGAAAGGACGAACATTCGGAAA | TCCGTCATCTCCATAGGGATCTT |
| mGAPDH | TGCACCACCAACTGCTTAG | GGATGCAGGGATGATGTTC |
| mCXCL-10 | GGATGGCTGTCCTAGCTCTG | TGAGCTAGGGAGGACAAGGA |

Supplement Table 1
